# The phylodynamics of the rabies virus in the Russian Federation

**DOI:** 10.1371/journal.pone.0171855

**Published:** 2017-02-22

**Authors:** Andrei A. Deviatkin, Alexander N. Lukashev, Elena M. Poleshchuk, Vladimir G. Dedkov, Sergey E. Tkachev, Gennadiy N. Sidorov, Galina G. Karganova, Irina V. Galkina, Mikhail Yu. Shchelkanov, German A. Shipulin

**Affiliations:** 1 Federal Budget Institute of Science Central Research Institute for Epidemiology, Moscow, Russian Federation; 2 Federal Budget Institute Chumakov Institute of Poliomyelitis and Viral Encephalitides, Moscow, Russian Federation; 3 Research Institute of Occupational Health, Moscow, Russian Federation; 4 Institute of Molecular Medicine, Sechenov First Moscow State Medical University, Moscow, Russia; 5 RUDN University, Moscow, Russia; 6 Institute for Natural Foci Infections, Omsk, Russian Federation; 7 Institute of Chemical Biology and Fundamental Medicine, Siberian Branch of the Russian Academy of Sciences (ICBFM SB RAS), Novosibirsk, Russian Federation; 8 Omsk State Pedagogical University, Omsk, Russian Federation; 9 Far Eastern Federal University, Vladivostok, Russian Federation; 10 Institute of Biology and Soil Science, Far Eastern Branch of Russian Academy of Sciences, Vladivostok, Russian Federation; National Institute of Health, ITALY

## Abstract

Near complete rabies virus N gene sequences (1,110 nt) were determined for 82 isolates obtained from different regions of Russia between 2008 and 2016. These sequences were analyzed together with 108 representative GenBank sequences from 1977–2016 using the Bayesian coalescent approach. The timing of the major evolutionary events was estimated. Most of the isolates represented the steppe rabies virus group C, which was found over a vast geographic region from Central Russia to Mongolia and split into three groups (C0-C2) with discrete geographic prevalence. A single strain of the steppe rabies virus lineage was isolated in the far eastern part of Russia (Primorsky Krai), likely as a result of a recent anthropogenic introduction. For the first time the polar rabies virus group A2, previously reported in Alaska, was described in the northern part of European Russia and at the Franz Josef Land. Phylogenetic analysis suggested that all currently circulating rabies virus groups in the Russian Federation were introduced within the few last centuries, with most of the groups spreading in the 20^th^ century. The dating of evolutionary events was highly concordant with the historical epidemiological data.

## Introduction

Rabies is a zoonotic viral disease, which is fatal once clinical manifestations develop. The classic rabies virus, RABV, is a negative-sense ssRNA virus belonging to the order *Mononegavirales*, the family *Rhabdoviridae* (from the Latin *rhabdos*, meaning “rod”, because representatives of this family have rod-shaped virions), and the genus *Lyssavirus* (Lyssa, an ancient Greek goddess, the personification of rabies). The genome of RABV consists of five protein-coding genes: N, P, M, G, and L. These genes are separated by intergenic regions of variable length. The N gene is commonly used for phylogenetic analysis [1].

Despite significant progress in fighting rabies worldwide [2] and successful eradication programs in Western Europe [3], rabies remains an important public health concern, causing more than two million disability-adjusted life years (DALYs) lost per year and with an annual economic cost of more than USD 4 billion per year [4]. Most human rabies cases (approximately 50,000 per year) occur in Africa and Asia [5]. It is assumed that the official statistical data is greatly underestimated due to the lack of systematic surveillance in some countries[6].

There were 22,264 animal rabies cases (49.0% wild animals, 30.0% domestic animals, 19.9% agricultural animals, 1.1% others) and 67 human rabies cases in the Russian Federation between 2007 and 2011. Around 400,000 people come into contact with potentially rabid animals and receive post-exposure prophylaxis each year [7, 8].

The genetic diversity of the rabies virus throughout the world has a strong geographic pattern, which results from the recent virus spread [9]. All currently known rabies viruses globally can be divided into seven major groups [10]. Two of these groups, the Cosmopolitan and the Arctic/Arctic-like, circulate in the Russian Federation. Within these two major groups, members of six smaller groups were reported in Russia [11–16]: A. Arctic rabies (northern parts of Siberia), B. Arctic-like rabies (Khabarovsk Krai, Transbaikal region), C. steppe rabies (Eurasian Steppe), D. Central European Russian rabies, E. Northeast European Rabies, and F. Caucasian rabies.

The aim of the current study was to investigate the phylodynamics of the rabies virus in the Russian Federation to gain a better understanding of virus spread patterns and timing.

Prior to this work, 227 unique rabies virus N-gene sequences from Russia (according to the sequence description) were available from GenBank and only 110 sequences were longer than 400 nt. We sequenced 81 strains from the collection at the Research Institute of Natural-Foci Infections (Omsk, Russia), almost doubling the number of available sequences. One more isolate was obtained from the Russian Arctic National Park in the Franz Josef Lands. Although the virus isolates were not systematically collected throughout the country, the dataset represented 18 out of 85 regions from both the European and Asian portions of Russia.

## Materials and methods

Near-complete N-gene sequences (1,110 nt) of the RABV isolates were identified by Sanger sequencing after a polymerase chain reaction (PCR) with oligonucleotides N1-F (ATGGATGCCGACAAGATTG)/N1-R (ATAGATGCTCAATCCGGGAG) and N2-F (ATGACAACTCACARAATGTGTGC)/N2-R(CCTCCATTCATCATGATTCG). Accession numbers are provided in Table A in S1 File.

For phylogenetic analysis, all available sequences with known collection dates containing positions 100 to 1,209 of the N-gene were used. Unfortunately, a significant number of sequences were unsuitable for Bayesian coalescent phylogenetic analysis due to an unknown collection date. Then, nearly identical sequences were excluded from the data set. In some cases, short or incompletely annotated sequences carried important epidemiological information. The phylogenetic position of such sequences was estimated by maximum likelihood reconstruction using MEGA 6.0 software (S1 Fig). The general time reversible (GTR) substitution model, incorporating a proportion of invariable sites (I) and a gamma distribution of rate variation among sites (G4), was found to be the best fit for the data according to the Akaike information criterion. These sequences are discussed in the text when necessary, but are not shown in the figures.

All available nucleotide sequences (as of July 4, 2016) which contained the words “rabies” and “nucleoprotein” in the definition field (12,051 sequences) were downloaded from GenBank. All sequences shorter than 1,110 nt, sequences without an isolation date, and incorrectly annotated sequences were removed. As only two of the major rabies virus groups circulate in Russia, we extracted all representatives of the Cosmopolitan group (nucleotide sequences that differed from GenBank entry KC538853 by no more than 6%) and the Arctic/Arctic-like group (which differed from GenBank entry KY002890 by no more than 9%). All sequences that differed from any other sequence in the dataset by less than 0.1% of the nucleotide sequence were omitted. Most sequences that belonged to groups that were not circulating in the Russian Federation were also excluded from further analysis. GenBank entry JQ685915 (North America bat RABV) was included as an outgroup. Sequences #FJ424484, U43433, U22475, U22840, JF973787, JF973796, KU198463, KU198471, KU198469, and U11375 were artificially added in order to broaden the data set.

The multiple sequence alignment was performed using MUSCLE as implemented in the MEGA 6.0 software [17]. The GenBank accession numbers for all RABV isolates analyzed in this study are presented in Table A in S1 File. The final data set consisted of 108 sequences.

The alignment was tested for recombination using the Recombination Detection Program (RDP) version 4 [18].

A reversible jump-based substitution model was used to choose the substitution model and estimate the appropriate number of parameters, while sampling the tree [19] was used for Bayesian coalescent analysis. Next, different clock assumptions and population models were compared by a Bayes factors test. The highest Bayes factor was observed in the combination of the uncorrelated lognormal relaxed clock and the constant population model, although there were no significant differences between the estimates for other combinations of two clock (strict and relaxed clock) and three population models (constant, exponential, and Bayesian skyline). The Markov chain Monte Carlo analysis was performed with 100 million generations (sampled every 10,000 generations).

The convergence of parameter estimates was checked using Tracer (v1.5) and indicated by an effective sample size > 200. The maximum clade credibility trees were annotated using TreeAnnotator (v2.4.2) after the starting 10% of trees were discarded. The resulting tree was visualized using FigTree (v1.4.2).

The visualization of the rabies virus distribution in the territory of the Russian Federation was performed with ArcGis (ESRI ArcGis version 10.4.1). Every point depicts the presence of the designated rabies virus group. Due to the lack of accurate geographical information, precision is limited by first-level administrative regions.

## Results

Prior to phylogenetic analysis, the data set was tested for recombination. Evidence for recombination in sequence U43432 was detected by the MaxChi method [20] and confirmed by phylogenetic reconstruction (data not shown). The relevance of this isolated finding is difficult to interpret. To avoid affecting the phylogenetic analysis, the sequence was excluded from the data set.

The mean nucleotide substitution rate, as inferred using the N-gene data set, was 2.47*10^−4^ [95% Highest Posterior Density (HPD) = 1.82–3.12*10^−4^] nucleotide substitutions/site/year. The rate is concordant with the previous estimate of RABV N-gene substitution rate, which was 2.3*10^−4^ [95% HPD = 1.1–3.6*10^−4^ substitutions/site/year] [9].

**Fig 1:**
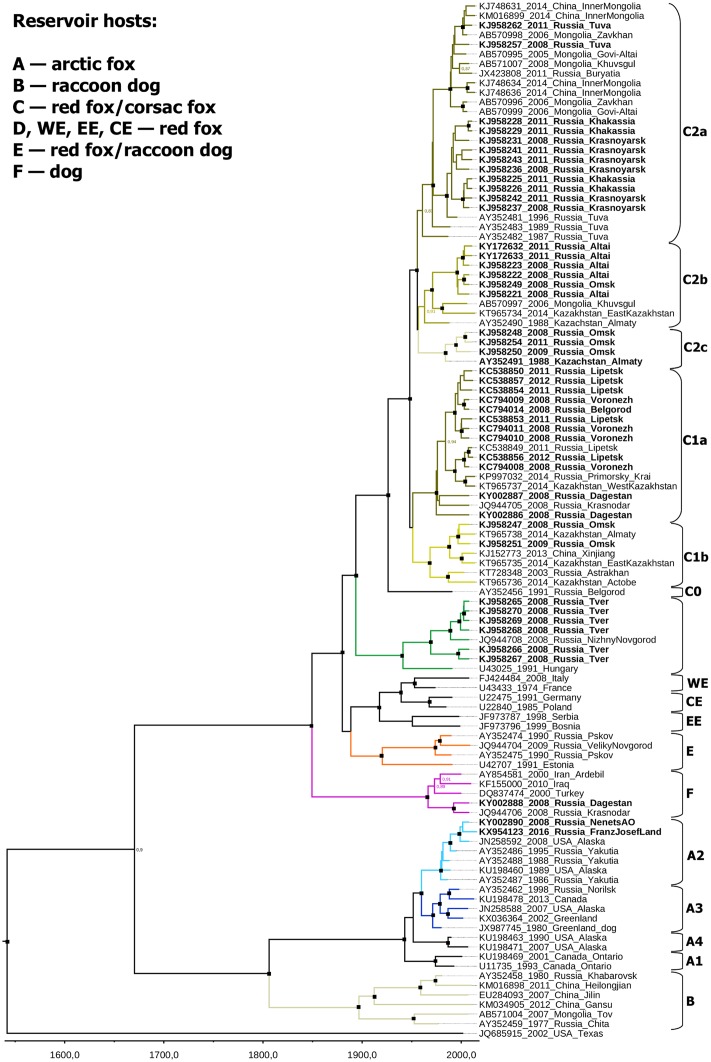
Bayesian phylogenetic analysis of near-complete N-gene sequences of the rabies virus strains circulating in the Russian Federation. Scale bar shows time in years. Branches are color-coded according to described groups. Node posterior probabilities above 95% are shown by black squares at the relevant nodes. Posterior probabilities between 80–95% are indicated by numbers. Posterior probabilities below 80% are not indicated. Sequences obtained in the current study are shown in bold.

Two major rabies virus lineages were circulating in the territory of the Russian Federation: Arctic/Arctic-like rabies (groups A and B according to the previously suggested classification [11]) and cosmopolitan rabies (groups C, D, E and F) (Fig 1).

Phylogenetic analysis revealed that the most recent common ancestor (MRCA) of all viruses currently circulating in Russian Federation existed in 1670 [1560–1782].

Groups C, D, E, and F (current Russian cosmopolitan rabies viruses) are descendants of the ancestor that most probably existed around 1849 [1796–1894]. These groups were not evenly represented in our data set.

Group D included seven isolates from the central European region of Russia. These isolates had a common ancestor in 1969 [1950–1986] and were closely related to a virus isolated in Hungary in 1991. The MRCA of these viruses and the Hungarian isolate dated back to 1941 [1915–1966]. Therefore, this group was likely introduced into Russia between 1915 and 1986 (extreme boundaries of the HPD intervals).

Most of the Russian isolates that were sequenced in this work (66/78) and most of the GenBank sequences from Russia in the final dataset (44/64) belonged to group C. One virus isolated from a cat in the Belgorod region in 1991 (AY352456) was from an outgroup within group C. We suggest that this strain should be considered as a representative of the independent rabies virus subgroup C0. The remaining viruses of group C were grouped together with a posterior probability of 1.0 and split into two subgroups (C1 and C2). The MRCA of these subgroups existed around 1948 [1929–1964].

**Fig 2:**
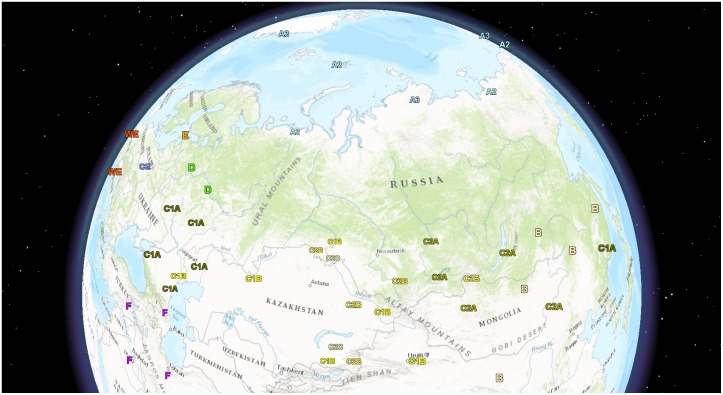
Distribution of rabies virus genotypes in the Russian Federation and neighboring countries. Geographical distribution of rabies virus phylogenetic groups is indicated. Precision is limited by the first-level administrative districts.

Subgroup C1 was not robustly supported (posterior probability 0.55) and could be alternatively defined as “all group C viruses that were not within C0 or C2 subgroups”. It included 23 viruses, mostly isolated to the west of the Tianshan and Altai mountains (Fig 2). The C1 subgroup could be subdivided into clusters C1a and C1b, which were supported with posterior probabilities of 1.0. The C1a cluster was prevalent in the steppe and the forest steppe ecological regions of the European part of Russia (Lipetsk, Voronezh, Belgorod, Krasnodar, Volgograd, Bryansk, Saratov regions, and the Dagestan Republic), in Kazakhstan (West Kazakhstan region), and in central and eastern regions of Ukraine (S1 fig). C1b cluster representatives were found in the Russian Omsk, Astrakhan, Orenburg, Bashkortostan, and Nizhny Novgorod regions, the Kazakhstan Aktobe, Almaty and East Kazakhstan provinces, and the Chinese Xinjiang Uyghur autonomous region.

Subgroup C2 (posterior probability 1.0) included 38 strains, mostly isolated to the east of the Astana-Tashkent line. There was a partial overlap in the distribution of subgroups C1 and C2 (Fig 2). The C2 subgroup split into three geographically discrete clusters. The first cluster (C2c) was found in the Omsk region (Russia) and the Almaty region (Kazakhstan), and the second one (C2a) was circulating in the territory of Southern Siberia (Buryatia, Khakassia, Tuva Republic, Omsk, and Krasnoyarsk regions), Kazakhstan (Almaty region), China (Inner Mongolia region), and Mongolia (Zavkhan, Govi-Altai, and Khuvsgul Provinces). Cluster C2b was prevalent in Russia (Altai Krai and Omsk regions), Mongolia (Khuvsgul Province), and Kazakhstan (Almaty and East Kazakhstan regions) (Figs 1 and 2). Posterior probability support for the C2a, C2b, and C2c clusters was 0.83, 0.91, and 1.0, respectively.

Group E included three viruses from the northwest part of Russia, which were closely related to the virus isolated in Estonia in 1991. Generally, group E corresponds to the previously described Northeast Europe group [21]. The MRCA of the Russian representatives of the Northeast Europe group existed around 1974 [1962–1983]. The MRCA of these viruses and the Estonian isolate existed in 1920 [1889–1950].

Group E (Northeast Europe) was closely related to the Western, Eastern, and Central Europe groups. The MRCA of the Northeast, Western, Eastern, and Central Europe groups existed around 1889 [1858–1924].

Group F (Caucasian rabies) included two viruses from the Krasnodar region and Dagestan. These viruses apparently originated from a common ancestor that existed around 1992 [1979–2004]. Other representatives of this group were found in the Middle East (Iraq, Iran and Turkey). The Iranian strain was previously described as belonging to the Iran-1a rabies virus phylogroup [22]. The MRCA of group F existed around 1966 [1947–1983].

Groups A (Arctic rabies) and B (Arctic-like rabies) diverged approximately in 1806 [1732–1871]. The Arctic rabies group A was previously subdivided into four subgroups: Arctic-1 (A1), Arctic-2 (A2), Arctic-3 (A3) and Arctic-4 (A4). Arctic-1 consists of strains only circulating in Ontario Province, Canada. Arctic-4 subgroup representatives were only found in the US state of Alaska. Arctic-2 and Arctic-3 subgroups are common over several vast territories: the northern part of Yakutia, Alaska (A2), the northern part of the Krasnoyarsk region, Canada, and Greenland (A3) [23–25]. According to our data the A2 subgroup emerged in 1979 [95% HPD 1973–1985], while the A3 subgroup emerged in 1971 [1962–1979]. The MRCA of A2 and A3 subgroups emerged approximately in 1960 [1946–1972]. These results are concordant with previous estimates using the N-gene [24]. However in another study [23], all timeframes were estimated to be significantly earlier. The cause of such disparity is unclear.

Interestingly, some representatives of the A2 subgroup (EF611834, EF611837, EF611840, EF611836, EF611832, EF611835, EF611839, and EF611838) [23] were isolated in the Yakutia region in 1950–1960 (as precise dates are unknown, these sequences were excluded from the Bayesian analysis). The estimated emergence of the current A2 subgroup seems to be correct because the modern rabies virus strains were apparently not descendants of this group, as represented by these archive isolates (data not shown). Apparently the Yakutian group became extinct, in concordance with the MRCA of current A2 subgroup representatives in 1979 (95% HPD 1973–1985).

In this work, representatives of the Arctic-2 subgroup were found in the northern part of the European region of Russia (the Nenetsk autonomous region, Komi Republic) and in the Franz Josef Land. Previously, no rabies cases had been reported in the Franz Josef Land. In 2016 during the monitoring of fallen animals in the Franz Josef Land nature resort, a carcass of an Arctic fox (*Vulpes lagopus*) was found in an unnatural pose. The rabies infection was confirmed by reverse transcriptase-PCR. The virus strain was subsequently isolated from the brain tissue and completely sequenced (excluding the 5’ and 3’ ends). The genome sequence was deposited in GenBank (accession number KX954123).

## Discussion

The principal objective of this study was to provide a comprehensive understanding of the circulation of the rabies virus in the Russian Federation. For the clarification of the phylogenetic relationship between different viral groups we used 64 sequences from 21 out of 85 administrative regions from both the European and Asian parts of Russia, and 45 sequences from 16 surrounding countries, which cover a time frame between 1977 and 2016.

Steppe rabies group C was the most widespread in the Russian Federation. It circulates in the steppe and forest steppe ecological regions of Eurasia and its principal reservoirs are foxes (*Vulpes vulpes* and *Vulpes corsac*). In our data set, group C was comprised of 61 viruses which were isolated over a large territory and diverged over the last 70 years from the MRCA that existed approximately in 1948. In the first half of the 20th century, rabies was registered predominantly in wolves, cats and dogs. The first cases of rabies in the Astrakhan region (Southern Russia) were registered in 1942 in a raccoon dog (*Nyctereutes procyonoides*). This was followed by a rabies outbreak in the fox population in the Astrakhan region in 1946 [26]. These were the first documented cases of rabies in foxes in the USSR [27]. According to historical statistical data, some authors assumed that the rapid expansion in the steppe rabies group started in the Astrakhan region, then spread to other territories with similar ecological conditions at an average rate of 40–60 km/year [28]. In 1949, fox rabies was registered in Kazakhstan. In Southern Siberia (Novosibirsk), the first fox rabies outbreaks occurred in 1958[11]. This historical rabies spread pattern and the hypothesis of subgroups C1/C2 originating in the early 1940s was supported by results of a Bayesian coalescent phylogenetic temporal analysis. The MRCA of steppe rabies subgroups C1/C2 was estimated to have existed in 1948 [1929–1964]. Furthermore, historic evidence of the rabies emergence in Novosibirsk in 1958 fits within the time frame of the probable MRCA of subgroup C2 in 1956 [1941–1970]. This subgroup includes viruses isolated in the Omsk and Krasnoyarsk regions, which border the Novosibirsk region from the west and the east, respectively.

Almost instantaneous spreading of the steppe rabies group in the vast Eurasian steppe and forest steppe occurred approximately in the 1940s and 1950s, and could reflect the so-called “Biological Big Bang” evolutionary model on a small scale [29,30]. This hypothesis implies that two qualitatively distinct phases exist in the evolutionary process: the inflationary phase (novel diverse groups are created) and the phase of slow evolution. In the 1940s and the 1950s, intense human migration in the territory of the former USSR occurred because of socioeconomic reasons. It is likely that animals carrying the steppe rabies virus ancestor were also transported and came into contact with the susceptible and naive local animal population. Introduction of the pathogen into a novel ecological niche (host shift to the fox population) could lead to the rapid spreading and diversification of the virus in the new territories (inflationary phase of evolution leading to the creation of geographically discrete mutant networks). As rabies is well known in all countries of the world, it is possible that similar expansions of the virus population occurred previously but left no trace due to the subsequent extinction of most virus variants.

The importance of hypothetical anthropogenic factors in the molecular evolution of rabies is highlighted by the isolation of the steppe C1a cluster in the far eastern part of Russia. Previously, circulation of only Arctic-like rabies virus strains (group B) was reported in the southern part of Russian’s Far East [11]. An attack of a brown bear (*Ursus arctos*) was registered in November 2014 in the Khasan region of Primorsky Krai (near the Pacific coast). The deviant behavior of the bear led to the suspicion that the animal was rabid. The disease was confirmed by several methods [31,32] and the virus was isolated and sequenced (accession number KP997032). Strikingly, this strain belonged to the Western steppe rabies C1a cluster. The most genetically related strains were isolated in the steppe regions of the European part of Russia (Lipetsk and Voronezh regions) and in the West Kazakhstan administrative region (Fig 1). For example, the far eastern bear isolate KP997032 and the KT965737 isolate from 2014 in the West Kazakhstan region share 99.67% nucleotide sequence identity in a 1,228 nt fragment of the N-gene. The MRCA of these two viruses existed in 2004 [1997–2012]. The distance between Primorsky Krai and Oral (the capital of the West Kazakhstan region) is 5,800 km. Other divergent members of the C group circulated in Southern Siberia, Kazakhstan, Mongolia, and the northwestern provinces of China (Fig 2). Therefore, anthropogenic virus transfer, which occurred less than 20 years ago, is the most probable mechanism for the emergence of the rabies virus group C in the southern part of Russia’s Far East.

In Europe the rabies virus evolution reportedly underwent two historical host shifts [21]. The first one occurred when the virus jumped from dogs to foxes, hypothetically in the early decades of the 20th century [33]. This suggested event corresponds to the common ancestor of groups WE, EE, CE and E (Western, Eastern, Central, and Northeastern Europe, respectively) on Fig 1. The inferred MRCA of these widespread European rabies virus subgroups existed around 1889 [1858–1924], concordant with the epidemiological hypothesis. The westward migration of rabid foxes from the former Soviet-Polish border has been registered since 1939. According to the molecular clock analysis, the MRCA of rabies virus strains isolated in Poland and Germany (Central Europe subgroup) and France and Italy (Western Europe subgroup) existed approximately in 1939 [1919–1958], which also corresponds to the field observations [34].

The second host switch took place in Northeastern Europe when the rabies virus colonized raccoon dogs. Between 1928 and 1957, approximately 9,100 animals were introduced from East Asia to more than 70 areas of the former USSR in an attempt to enrich the fauna with a commercial fur animal [35]. Raccoon dogs successfully colonized western regions of the former USSR. Today, this alien species has invaded a large part of Europe (from Russia in the east to Germany in the west, from Finland in the north to Slovakia and Moldova in the south). Rabies virus group E (NEE according to another classification [11]) is maintained in two main wildlife reservoirs: foxes and raccoon dogs. According to our estimates, the MRCA of group E existed around 1920 [1889–1950]. Previously it was unclear whether the source of the virus in raccoon dogs was infected foxes or a virus that jumped to raccoon dogs directly from dogs and was then passed onto the fox population [21]. According to the molecular analysis, the ancestral virus population of modern group E viruses could exist in Europe before the appearance of raccoon dogs; however a wide HPD interval at this node precludes a conclusive answer.

Caucasian rabies (group F) belonged to the earlier described Iran-1a group [22]. It is the canine rabies group that was prevalent in the Middle East region (Iraq, Iran, and Turkey). The first Russian representatives of this group were isolated in 2005 [12]. In addition, the deepest branches within group F were found in the Middle East. Therefore, it can be assumed that this virus group was not circulating in Russia before 1979 (lower extreme boundary of the HPD interval for the MRCA of Russian representatives of group F) and was likely more recently introduced into Russia from the bordering Caucasian states. Meanwhile, rabies cases were detected in Caucasian regions of the Russian Federation much earlier, although the sequences of these viruses are not available. As a result, the comprehensive history of group F in the territory of Russia remains unknown.

Arctic rabies subgroups A2 and A3 are distributed over vast circumpolar territories, but their genetic diversity is extremely low compared to other virus groups. For example, the N-gene of KX954123 (isolated on the Franz Josef Land, Russia, in 2016) shares 99.3% identical nucleotides with the N-gene of JN258592 (isolated in Alaska in 2008). All strains of A2 and A3 subgroups shared 97.39–100.00% sequence identity. Such low diversity can be explained by rapid virus spread due to long-distance migration of polar carnivores. Indeed, in March the Arctic ice area is at its maximum and all circumpolar territories (Alaska, Northern Canada, Greenland, and Northern Russia) represent a united ecological region (S2 fig) with similar environmental conditions. The principal host of Arctic rabies is the Arctic fox (*Vulpes lagopus*), which is able to migrate more than 5,000 km during the cold season [36]. Here we described the virus that was found at the Franz Joseph Land and was most similar to the Alaskan but not the North Siberian isolates, which further illustrates the high spatial spread rate of the polar rabies.

Rabies is transmitted mainly by direct physical contact. Therefore, a sufficient density of animal population is required for efficient transmission [34]. Animal population density fluctuates constantly due to changing environmental conditions, which may result in the extinction of rabies virus phylogenetic groups. However, after the host density decreases below the critical level and the virus becomes extinct, animal populations usually recover and become susceptible to rapid virus spread. Our analysis supports this model, which implies virus population expansion and extinction cycles and has important practical implications. There are two possible ways to eliminate the rabies virus. This could be accomplished by either reducing the density of principal hosts by partial extermination of the animal population, or by reducing the density of susceptible principal hosts by oral vaccination of the animal population. Attempts to control the spread of rabies in Europe by host population reduction have proven ineffective [34]. However, implementation of oral rabies vaccination (ORV) programs in Western European countries (S3 Fig) has proven to be the most effective method to eliminate rabies [37]. Previous unsuccessful attempts questioned the feasibility of rabies eradication in Russia using ORV [38], although this failure could be attributed to poor organization [39].

Phylogenetic analysis suggested a relatively recent emergence of all rabies virus groups currently circulating in the territory of the Russian Federation. Meanwhile, rabies was known in Russia much earlier [40]. Therefore, the ancient virus variants became extinct due to natural reasons and the virus population was not stable in the long term. This conclusion implies that pressure on the virus population provided by the oral vaccination of wild carnivores can result in virus extinction. However, further field studies are required for the formulation of an effective country-wide rabies elimination strategy. At the same time, some reservoirs of the rabies virus, such as bats, cannot be involved in vaccination programs [41]. Therefore, even in the case of carnivore RABV elimination, the global eradication of rabies and rabies-like viruses does not seem feasible due to the probability of cross-species spillover of infection [42].

## Conclusion

The rabies eradication program was successful in Western Europe, yet rabies remains endemic in Russia. Analysis indicates that specific virus groups circulate in distinct ecological regions. The virus population is surprisingly simple and suggests a straightforward spread pattern, which apparently occurred over the last few centuries with the absence of long-term virus endemicity. Only a few virus introductions from neighboring countries were evident. Such a pattern implies that the elimination of rabies over major parts of Russia is feasible.

## Supporting information

S1 Fig Maximum likelihood phylogenetic tree of the extended rabies virus N-gene sequences data set. (TIF)

S2 Fig The average sea ice extent in September 2015 (left) and March 2015 (right) illustrate the respective minimum and maximum extents. The magenta line indicates the median ice extents in September and March, respectively, during the period 1981 to 2010. Maps were obtained from the NSIDC (National Snow and Ice Data Center) at http://www.nsidc.org/data/seaice_index.
(TIF)

S3 Fig **(A) Reported mammalian rabies cases in 1991 (excluding bat rabies). (B) Reported mammalian rabies cases in 2014 (excluding bat rabies). (C) Wild animal ORV campaigns in 2005–2014 [Rabies Information System of the WHO Collaboration Centre for Rabies Surveillance and Research,** http://www.who-rabies-bulletin.org/Queries/Maps.aspx].
(TIF)

S1 File (Table A) Rabies virus nucleoprotein sequences used in this study. (DOC)
