# Supplementary figures and images for: The phylodynamics of the rabies virus in the Russian Federation
Source: PLoS One. 2017 Feb 22;12(2):e0171855. doi: 10.1371/journal.pone.0171855 (PMC5321407; doi:10.1371/journal.pone.0171855)

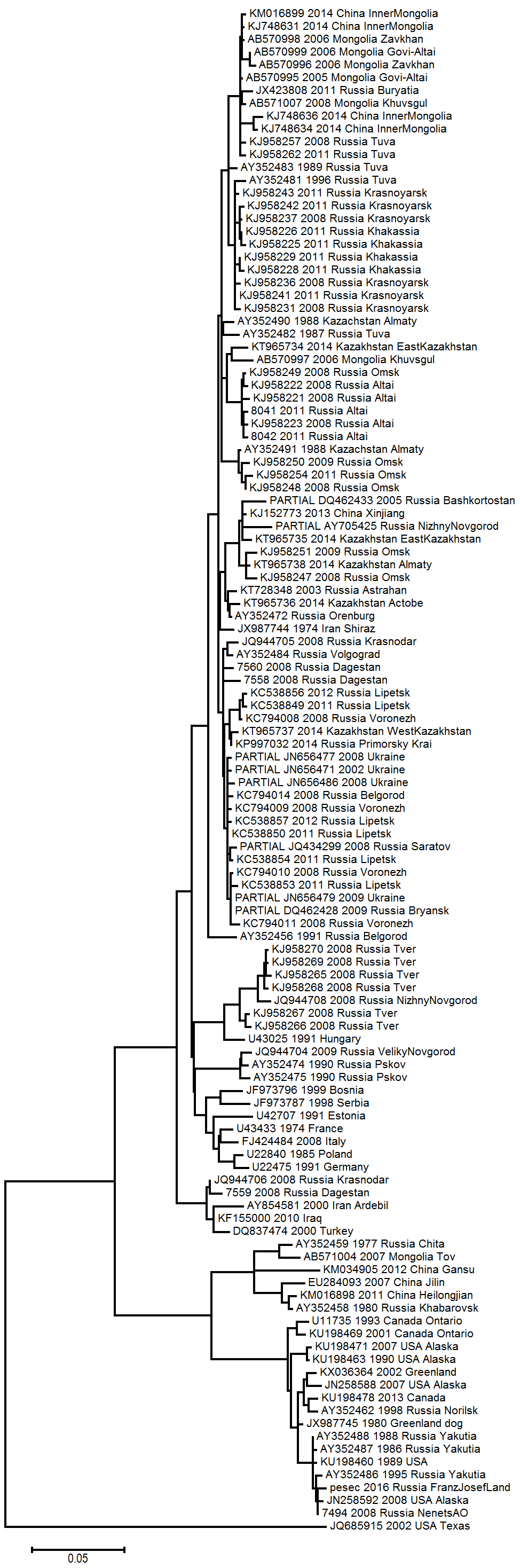

Supplement: S1 Fig — (TIF) [file pone.0171855.s001.tif]

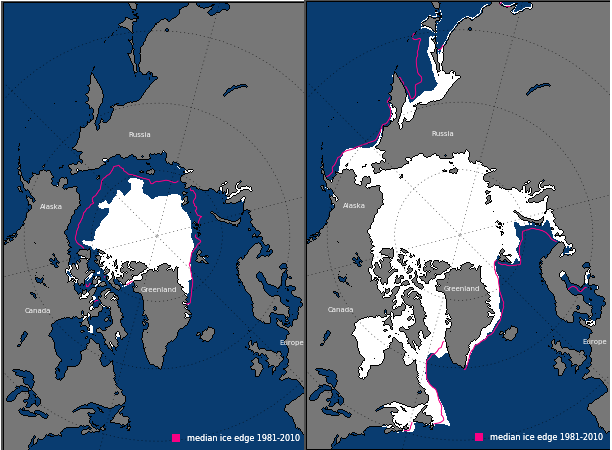

Supplement: S2 Fig — The magenta line indicates the median ice extents in September and March, respectively, during the period 1981 to 2010. Maps were obtained from the NSIDC (National Snow and Ice Data Center) at http://www.nsidc.org/data/seaice_index. (TIF) [file pone.0171855.s002.tif]

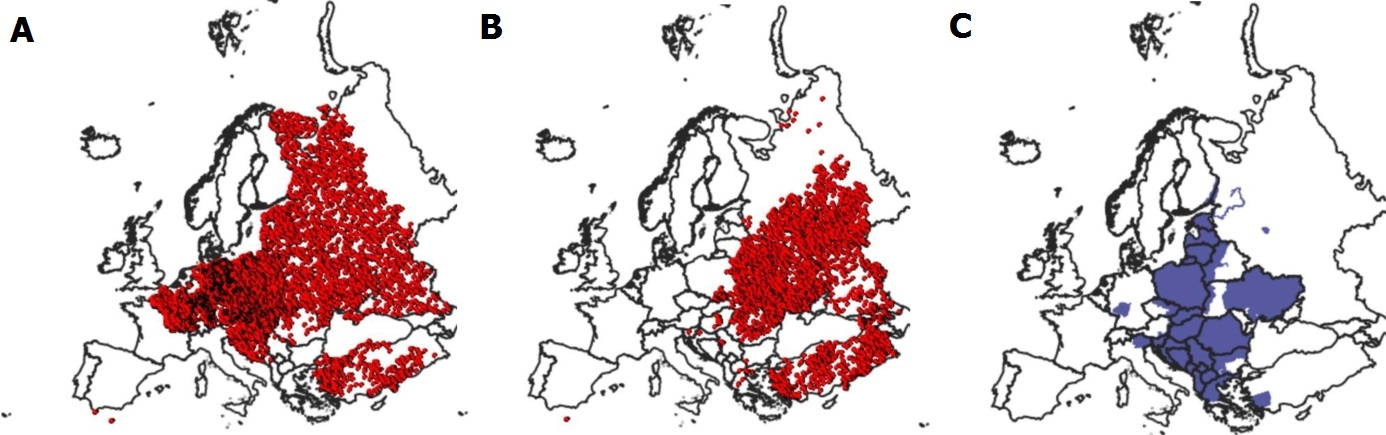

Supplement: S3 Fig — (A) Reported mammalian rabies cases in 1991 (excluding bat rabies). (B) Reported mammalian rabies cases in 2014 (excluding bat rabies). (C) Wild animal ORV campaigns in 2005–2014 [Rabies Information System of the WHO Collaboration Centre for Rabies Surveillance and Research, http://www.who-rabies-bulletin.org/Queries/Maps.aspx]. (TIF) [file pone.0171855.s003.tif]
