# Supplementary material for: The phylodynamics of the rabies virus in the Russian Federation
Source: PLoS One. 2017 Feb 22;12(2):e0171855. doi: 10.1371/journal.pone.0171855 (PMC5321407; doi:10.1371/journal.pone.0171855)
Supplement: S1 File — (DOC) [file pone.0171855.s004.doc]

**Table A**. Rabies virus nucleoprotein sequences used in this study.

| Accession number | Place of origin | Year of isolation | Reference |
| --- | --- | --- | --- |
| KC538861 | Russia, Lipetsk | 2012 | This study |
| KC538860 | Russia, Lipetsk | 2012 | This study |
| KC538859 | Russia, Lipetsk | 2012 | This study |
| KC538858 | Russia, Lipetsk | 2012 | This study |
| KC538857 | Russia, Lipetsk | 2012 | This study |
| KC538856 | Russia, Lipetsk | 2012 | This study |
| KC538855 | Russia, Lipetsk | 2012 | This study |
| KC538854 | Russia, Lipetsk | 2012 | This study |
| KC538853 | Russia, Lipetsk | 2012 | This study |
| KC538852 | Russia, Lipetsk | 2011 | This study |
| KC538851 | Russia, Lipetsk | 2011 | This study |
| KC538850 | Russia, Lipetsk | 2011 | This study |
| KC538849 | Russia, Lipetsk | 2011 | This study |
| KC794014 | Russia, Belgorod | 2008 | This study |
| KC794013 | Russia, Belgorod | 2008 | This study |
| KC794012 | Russia, Belgorod | 2008 | This study |
| KC794011 | Russia, Voronezh | 2008 | This study |
| KC794010 | Russia, Voronezh | 2008 | This study |
| KC794009 | Russia, Voronezh | 2008 | This study |
| KC794008 | Russia, Voronezh | 2008 | This study |
| KC794007 | Russia, Voronezh | 2008 | This study |
| KJ958270 | Russia, Tver | 2008 | This study |
| KJ958269 | Russia, Tver | 2008 | This study |
| KJ958268 | Russia, Tver | 2008 | This study |
| KJ958267 | Russia, Tver | 2008 | This study |
| KJ958266 | Russia, Tver | 2008 | This study |
| KJ958265 | Russia, Tver | 2008 | This study |
| KJ958264 | Russia, Tuva | 2012 | This study |
| KJ958263 | Russia, Tuva | 2012 | This study |
| KJ958262 | Russia, Tuva | 2011 | This study |
| KJ958261 | Russia, Tuva | 2010 | This study |
| KJ958260 | Russia, Tuva | 2008 | This study |
| KJ958259 | Russia, Tuva | 2008 | This study |
| KJ958258 | Russia, Tuva | 2008 | This study |
| KJ958257 | Russia, Tuva | 2008 | This study |
| KJ958256 | Russia, Tuva | 2008 | This study |
| KJ958255 | Russia, Omsk | 2011 | This study |
| KJ958254 | Russia, Omsk | 2011 | This study |
| KJ958253 | Russia, Omsk | 2010 | This study |
| KJ958252 | Russia, Omsk | 2010 | This study |
| KJ958251 | Russia, Omsk | 2009 | This study |
| KJ958250 | Russia, Omsk | 2009 | This study |
| KJ958249 | Russia, Omsk | 2008 | This study |
| KJ958248 | Russia, Omsk | 2008 | This study |
| KJ958247 | Russia, Omsk | 2008 | This study |
| KJ958246 | Russia, Krasnoyarsk | 2011 | This study |
| KJ958245 | Russia, Krasnoyarsk | 2011 | This study |
| KJ958244 | Russia, Krasnoyarsk | 2011 | This study |
| KJ958243 | Russia, Krasnoyarsk | 2011 | This study |
| KJ958242 | Russia, Krasnoyarsk | 2011 | This study |
| KJ958241 | Russia, Krasnoyarsk | 2011 | This study |
| KJ958240 | Russia, Krasnoyarsk | 2011 | This study |
| KJ958239 | Russia, Krasnoyarsk | 2011 | This study |
| KJ958238 | Russia, Krasnoyarsk | 2008 | This study |
| KJ958237 | Russia, Krasnoyarsk | 2008 | This study |
| KJ958236 | Russia, Krasnoyarsk | 2008 | This study |
| KJ958235 | Russia, Krasnoyarsk | 2008 | This study |
| KJ958234 | Russia, Krasnoyarsk | 2008 | This study |
| KJ958233 | Russia, Krasnoyarsk | 2008 | This study |
| KJ958232 | Russia, Krasnoyarsk | 2008 | This study |
| KJ958231 | Russia, Krasnoyarsk | 2008 | This study |
| KJ958230 | Russia, Khakassia | 2011 | This study |
| KJ958229 | Russia, Khakassia | 2011 | This study |
| KJ958228 | Russia, Khakassia | 2011 | This study |
| KJ958227 | Russia, Khakassia | 2011 | This study |
| KJ958226 | Russia, Khakassia | 2011 | This study |
| KJ958225 | Russia, Khakassia | 2011 | This study |
| KJ958224 | Russia, Altai | 2008 | This study |
| KJ958223 | Russia, Altai | 2008 | This study |
| KJ958222 | Russia, Altai | 2008 | This study |
| KJ958221 | Russia, Altai | 2008 | This study |
| KX954123 | Russia, Franz Joseph Land | 2016 | This study |
| KY002886 | Russia, Dagestan | 2008 | This study |
| KY002887 | Russia, Dagestan | 2008 | This study |
| KY002888 | Russia, Dagestan | 2008 | This study |
| KY002889 | Russia, Dagestan | 2008 | This study |
| KY002890 | Russia, Nenetsk autonomous region | 2008 | This study |
| KY002891 | Russia, Nenetsk autonomous region | 2008 | This study |
| KY002892 | Russia, Nenetsk autonomous region | 2008 | This study |
| KY002893 | Russia, Nenetsk autonomous region | 2008 | This study |
| KY172632 | Russia, Altai | 2011 | This study |
| KY172633 | Russia, Altai | 2011 | This study |
| AB571004 | Mongolia, Tov | 2007 | [43] |
| AB570999 | Mongolia, Govi-Altai | 2006 | [43] |
| AB570998 | Mongolia, Zavkhan | 2005 | [43] |
| AB570997 | Mongolia, Khuvsgul | 2006 | [43] |
| AB570996 | Mongolia, Zavkhan | 2006 | [43] |
| AB570995 | Mongolia, Govi-Altai | 2005 | [43] |
| AB571007 | Mongolia, Khuvsgul | 2008 | [43] |
| KM016899 | China, Inner Mongolia Autonomous Region | 2014 | [44] |
| KJ748631 | China, Inner Mongolia Autonomous Region | 2014 | [45] |
| KJ748634 | China, Inner Mongolia Autonomous Region | 2014 | [45] |
| KJ748636 | China, Inner Mongolia Autonomous Region | 2014 | [45] |
| KJ152773 | China, Xinjiang Autonomous Region and | 2013 | [45] |
| AY352481 | Russia, Tuva | 1996 | [11] |
| AY352482 | Russia, Tuva | 1989 | [11] |
| AY352483 | Russia, Tuva | 1987 | [11] |
| AY352490 | Kazakhstan, Almaty | 1988 | [11] |
| AY352491 | Kazakhstan, Almaty | 1988 | [11] |
| AY352456 | Russia, Belgorod | 1991 | [11] |
| AY352474 | Russia, Pskov | 1990 | [11] |
| AY352475 | Russia, Pskov | 1990 | [11] |
| AY352486 | Russia, Yakutia | 1995 | [11] |
| AY352487 | Russia, Yakutia | 1986 | [11] |
| AY352488 | Russia, Yakutia | 1988 | [11] |
| AY352462 | Russia, Norilsk | 1998 | [11] |
| AY352458 | Russia, Khabarovsk | 1980 | [11] |
| AY352459 | Russia, Chita | 1977 | [11] |
| JX423808 | Russia, Buryatia | 2011 | [46] |
| KT965734 | Kazakhstan, East Kazakhstan | 2014 | Not published |
| KT965735 | Kazakhstan, East Kazakhstan | 2014 | Not published |
| KT965736 | Kazakhstan, Actobe | 2014 | Not published |
| KT965737 | Kazakhstan, West Kazakhstan | 2014 | Not published |
| KT965738 | Kazakhstan, Almaty | 2014 | Not published |
| KP997032 | Russia, Primorsky krai | 2014 | [31] |
| JQ944705 | Russia, Nizhny Novgorod | 2008 | [16] |
| JQ944706 | Russia, Krasnodar | 2008 | [16] |
| JQ944708 | Russia, Krasnodar | 2008 | [16] |
| KT728348 | Russia, Astrakhan | 2003 | [47] |
| JX987744 | Iran, Shiraz | 1974 | [22] |
| U43025 | Hungary | 1991 | [21] |
| U43433 | France | 1974 | [21] |
| U22475 | Germany | 1991 | [48] |
| U22840 | Poland | 1985 | [48] |
| U42707 | Estonia | 1991 | [21] |
| FJ424484 | Italy | 2008 | [49] |
| JF973787 | Serbia | 1998 | [33] |
| JF973796 | Bosnia | 1998 | [33] |
| AY854581 | Iran, Ardebil | 2000 | [22] |
| KF155000 | Iraq | 2010 | [50] |
| DQ837474 | Turkey | 2000 | [51] |
| JN258592 | USA, Alaska | 2008 | [24] |
| KU198460 | USA, Alaska | 1989 | [25] |
| KU198478 | Canada, Nunavut | 2013 | [25] |
| JN258588 | USA, Alaska | 2007 | [24] |
| KX036364 | Greenland | 2002 | [25] |
| KU198471 | USA, Alaska | 2007 | [25] |
| KU198463 | USA, Alaska | 1990 | [25] |
| KU198469 | Canada, Ontario | 2001 | [25] |
| U11735 | Canada, Ontario | 1993 | Not published |
| KM016898 | China, Heilongjian | 2011 | [44] |
| EU284093 | China, Jilin | 2007 | [52] |
| KM034905 | China, Gansu | 2012 | [53] |
| JQ685915 | USA, Texas | 2002 | [54] |

References

43. Boldbaatar B, Inoue S, Tuya N, et al. Molecular epidemiology of rabies virus in Mongolia, 2005-2008. Jpn J Infect Dis. 2010;63(5):358-363.

44. Liu Y, Zhang S, Zhao J, et al. Fox- and raccoon-dog-associated rabies outbreaks in northern China. Virol Sin. 2014;29(5):308-310. doi:10.1007/s12250-014-3484-0.

45. Feng Y, Wang W, Guo J, et al. Disease outbreaks caused by steppe-type rabies viruses in China. EpidemiolInfect. 2014;143:1287-1291. doi:10.1017/S0950268814001952.

46. Adelshin R V., Melnikova O V., Trushina YN, et al. A new outbreak of fox rabies at the Russian-Mongolian border. Virol Sin. 2015;30(4):313-315. doi:10.1007/s12250-015-3609-0.

47. Dedkov VG, Lukashev AN, Deviatkin AA, et al. Retrospective diagnosis of two rabies cases in humans by high throughput sequencing. J Clin Virol. 2016;78:74-81. doi:10.1016/j.jcv.2016.03.012.

48. Kissi B, Tordo N, Bourhy H. Genetic polymorphism in the rabies virus nucleoprotein gene. Virology. 1995;209:526-537. doi:10.1006/viro.1995.1285.

49. De Benedictis P, Gallo T, Iob A, et al. Emergence of fox rabies in north-eastern Italy. Euro Surveill Bull Eursur les Mal Transm = Eur Commun Dis Bull. 2008;13(45):6-7.

50. Marston DA, McElhinney LM, Ellis RJ, et al. Next generation sequencing of viral RNA genomes. BMC Genomics. 2013;14(1):444. doi:10.1186/1471-2164-14-444.

51. David D, Hughes GJ, Yakobson B a, et al. Identification of novel canine rabies virus clades in the Middle East and North Africa. J Gen Virol. 2007;88(Pt 3):967-980. doi:10.1099/vir.0.82352-0.

52. Shao XQ, Yan XJ, Luo GL, et al. Genetic evidence for domestic raccoon dog rabies caused by Arctic-like rabies virus in Inner Mongolia, China. Epidemiol Infect. 2011;139:629-635. doi:10.1017/S0950268810001263.

53. Tao XY, Guo ZY, Li H, et al. Rabies Cases in the West of China Have Two Distinct Origins. PLoS Negl Trop Dis. 2015;9(10):1-8. doi:10.1371/journal.pntd.0004140.

54. Kuzmin I V., Shi M, Orciari LA, et al. Molecular inferences suggest multiple host shifts of rabies viruses from bats to mesocarnivores in Arizona during 2001-2009. PLoS Pathog. 2012;8(6). doi:10.1371/journal.ppat.1002786
